# Induction of the HIV-1 Tat co-factor cyclin T1 during monocyte differentiation is required for the regulated expression of a large portion of cellular mRNAs

**DOI:** 10.1186/1742-4690-3-32

**Published:** 2006-06-09

**Authors:** Wendong Yu, Yan Wang, Chad A Shaw, Xiao-Feng Qin, Andrew P Rice

**Affiliations:** 1 Department of Molecular Virology and Microbiology, Baylor College of Medicine, Houston, Texas 77030, USA; 2 Department of Human and Molecular Genetics, Baylor College of Medicine, Houston, Texas, USA; 3 Center for Cancer Immunology Research, Department of Immunology, The University of Texas M.D. Anderson Cancer Center, Houston, Texas, USA

## Abstract

**Background:**

P-TEFb, a general RNA polymerase II elongation factor, is composed of CDK9 (cyclin-dependent kinase 9) as a catalytic unit and either cyclin T1, T2 or K as a regulatory subunit. The cyclin T1/P-TEFb complex is targeted by HIV to mediate Tat transactivation. Cyclin T1 protein expression is induced during early macrophage differentiation, suggesting a role in regulation of mRNA expression during the differentiation process. To study the functional significance of cyclin T1 induction during differentiation, we utilized the human Mono Mac 6 (MM6) monocytic cell line.

**Results:**

We found that cyclin T1 protein expression is induced by a post-transcriptional mechanism following PMA treatment of MM6 cells, similar to its induction in primary monocytes and macrophages. Also in agreement with findings in primary cells, cyclin T2a is present at relatively high levels in MM6 cells and is not induced by PMA. Although the knock-down of cyclin T1 in MM6 cells by shRNA inhibited HIV-1 Tat transactivation, MM6 cell growth was not affected by the depletion of cyclin T1. Using DNA microarray technology, we found that more than 20% of genes induced by PMA require cyclin T1 for their normal level of induction, and approximately 15% of genes repressed by PMA require cyclin T1 for their normal level of repression. Gene ontology analysis indicates that many of these cyclin T1-dependent genes are related to immune response and signal transduction.

**Conclusion:**

These results suggest that cyclin T1 serves a critical role in the program of macrophage differentiation, and this raises questions about the feasibility of cyclin T1 serving as an antiviral therapeutic target.

## Background

Mammalian RNA polymerase II transcription (RNAP II) is a complex and coordinated process and its regulation is involved in many important cellular events such as differentiation, activation, and stress response. While the regulation of transcription initiation has been an actively studied area for decades, the regulation of transcription elongation has not been as actively investigated until recent years when a number of transcription elongation factors have been identified [1]. One factor of particular interest to transcriptional elongation is P-TEFb, a protein kinase that appears to regulate expression of a large portion of mammalian genes [2,3]. P-TEFb is believed to activate transcriptional elongation through phosphorylation of the carboxyl-terminal domain of RNAP II, the Spt5 subunit of the DSIF complex, and the RD subunit of the NELF complex, therefore overcoming blocks to RNAP II processivity [4-6].

A number of distinct P-TEFb complexes exist in human cells. All P-TEFb complexes contain CDK9 as the catalytic subunit, either the major 42 kDa CDK9 protein or the 55 kDa CDK9 protein, a minor isoform containing an amino terminal extension that arises from an upstream transcriptional start site [7]. These CDK9 proteins are associated with a regulatory cyclin subunit, which can be either cyclin T1, T2a, T2b, or cyclin K [8]. The existence of different P-TEFb complexes raises the possibility that distinct sets of genes may be regulated by different P-TEFb complexes. Consistent with this idea, the CDK9 42 kDa protein is localized throughout the nucleoplasm, while the CDK9 55 kDa protein is concentrated in the nucleolus [9]. Additionally, the 55 kDa protein is expressed at relatively high levels in resting lymphocytes and is not regulated by activation, while the 42 kDa protein is expressed at low levels in resting lymphocytes and is upregulated by activation [9]. Additionally, a large portion of P-TEFb is associated in a large complex containing 7SK snRNA and HEXIM proteins, either HEXIM I or HEXIM II [10-15]. This large P-TEFb is catalytically inactive *in vitro *and it has been proposed that 7SK snRNA and HEXIM proteins are negative regulators of transcription elongation.

The best-characterized P-TEFb complex is cyclin T1/CDK9, which is targeted by the human immunodeficiency virus-1 (HIV-1) Tat protein to stimulate the transcription elongation and therefore the replication of the integrated HIV-1 genome [16,17]. Because of its important role in HIV-1 replication, the inhibition of P-TEFb function has been proposed as a potential therapeutic approach for AIDS. Thus far, proposed methods of inhibiting P-TEFb function include: small molecule inhibitors, anti-hCycT1 intrabodies, a dominant-negative CDK9 protein, and siRNAs against P-TEFb [18-23].

In human monocytes and macrophages, primary targets of HIV-1 infection, we have previously observed complex patterns of P-TEFb regulation. Cyclin T1 mRNA levels are high but little protein expression can be observed in monocytes freshly isolated from health blood donors [24]. When monocytes are cultured under conditions that induce macrophage differentiation, cyclin T1 protein expression is induced to high levels within one to two days. In contrast, CDK9 protein levels are generally high in freshly isolated monocytes and are not strongly upregulated during differentiation. However, after approximately seven to ten days of macrophage differentiation in culture, cyclin T1 protein expression is shut-off by proteasome-mediated proteolysis that may target the PEST sequence at the carboxyl terminus of cyclin T1 [25]. Macrophage activators such as lipopolysacchride or other pathogen-associated molecular patterns (PAMPs) can reinduce expression of cyclin T1 after the shut-off, suggesting that induction of cyclin T1 is a component of an innate immune response [25]. Interestingly, HIV infection can also induce cyclin T1 expression in the late-differentiated macrophages [25]. In contrast to the regulated expression of cyclin T1, the cyclin T2a subunit of P-TEFb is present at relatively high levels in monocytes, it is not shut off during differentiation, and it is not induced by activation [26]. These data suggest that cyclin T2a and T1 might regulate the expression of different genes in monocytes and macrophages. Moreover, the expression pattern of cyclin T1 suggests that it may specifically regulate genes important for macrophage early differentiation and the innate immune response.

In this study, we report that in a monocytic cell line, Mono Mac 6 (MM6), cyclin T1 protein expression is induced by a post-transcriptional mechanism following PMA treatment to induce macrophage differentiation, similar to the induction of cyclin T1 in primary monocytes and macrophages. Also similar to primary cells, cyclin T2a is present at relative high levels in MM6 cells and is not responsive to differentiation signals. We found that although knock-down of cyclin T1 in MM6 cells by shRNA inhibits HIV-1 Tat transactivation, it did not affect cell growth. Using DNA microarray technology, we found that the knock-down of cyclin T1 had a relatively small effect on mRNA levels in MM6 cells prior to PMA treatment, consistent with no obvious effect of the knock-down on cell growth. However, more than 20% of genes induced by PMA require cyclin T1 for their normal level of induction, and approximately 15% of genes repressed by PMA require cyclin T1 for their normal level of repression. These results suggest that cyclin T1 serves a critical role in the PMA-induced program of macrophage differentiation of MM6 cells. Therefore, the use of cyclin T1 as an antiviral therapeutic target may not be feasible.

## Results

### Establishment of a model system for investigation of cyclin T1 function in macrophage differentiation

**Figure 1:**
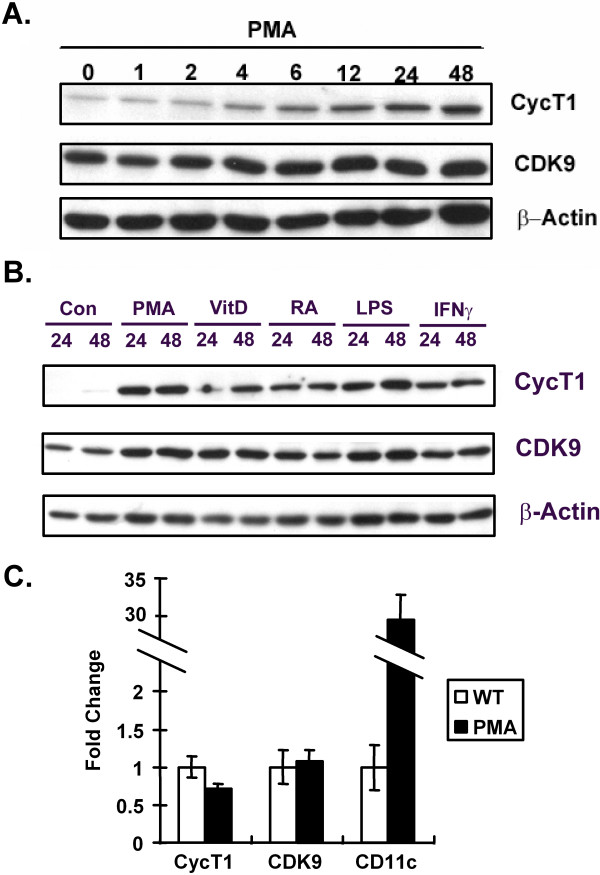
**Cyclin T1 expression is induced in MM6 cells through a post-transcriptional mechanism**. (A) Cell extracts were prepared from untreated MM6 cells or MM6 cells treated with PMA from 1 to 48 hours as indicated. Immunoblots were performed to measure levels of Cyclin T1 (CycT1), CDK9 and β-actin proteins. (B) Cell extracts were prepared from untreated MM6 cells (Con) or MM6 cells treated with PMA, vitamin D3 (VitD), retinoic acid (RA), LPS, or interferon gamma (IFNγ) for 24 or 48 hours. Immunoblots were performed to measure levels of Cyclin T1, CDK9 and β-actin proteins. (C) Total RNA was isolated from untreated MM6 cells or cells treated with PMA for 24 hours. Quantitative real-time RT-PCR was used to measure the expression level of Cyclin T1, CDK9, and CD11c mRNA. The fold-change represents the change of transcript levels in PMA-treated MM6 cells relative to untreated cells after normalization to β-actin mRNA levels which are insensitive to PMA.

The functional significance of the induction of cyclin T1 expression upon differentiation of primary monocytes is unknown, in part due to the difficulty in biochemical and genetic manipulation of primary monocytes. To determine whether the induction of cyclin T1 protein can be recapitulated in a transformed cell line that is more amenable to functional studies, we examined the Mono-Mac-6 (MM6) cell line that was derived from a human leukemia patient [27]. MM6 cells exhibits characteristics of mature monocytes, such as the expression of markers specific for mature monocytes which are absent in the less mature and more commonly used U937 and THP1 human promonocytic cell lines [27]. To examine cyclin T1 expression in MM6 cells, a time-course experiment was performed in MM6 cells using PMA treatment as the differentiation agent (Fig. 1A). Following 24 hours of PMA treatment, MM6 cells aggregated and became loosely attached to the bottom of the culture dishes (data not shown), mimicking the differentiation of monocytes into macrophages. Cyclin T1 expression was low prior to the treatment and an induction of its expression was observed as early as six hours after PMA treatment and continued to increase at 24 and 48 hours. In contrast, CDK9 and β-actin were expressed at relatively constant high levels before and after PMA treatment (Fig. 1A).

To determine whether the cyclin T1 induction in MM6 cells is specific to PMA, other differentiation inducers or macrophage activators were tested for their effect on cyclin T1 expression (Fig. 1B). Treatment of MM6 cells with the differentiation inducers vitamin D3 or retinoic acid showed strong induction of cyclin T1 at 24 and 48 hours post-treatment, similar to that of PMA. Treatment of MM6 cells with the activators LPS or interferon-γ also showed a strong induction of cyclin T1 at 24 and 48 hours post-treatment (Fig. 1B).

The expression of cyclin T1 in primary macrophages is known to be regulated post-transcriptionally, as the mRNA for cyclin T1 is high in primary monocytes when cyclin T1 protein expression is low and it does not increase with the induction of cyclin T1 protein expression [24]. To examine whether the induction of cyclin T1 in MM6 cells is also regulated by a post-transcriptional mechanism, the mRNA expression levels of cyclin T1 were examined by quantitative RT-PCR analysis (Fig. 1C). Although cyclin T1 protein expression was induced by PMA (data not shown), the mRNA level of cyclin T1 did not increase after the treatment of PMA and actually decreased about 40%. This reduction in cyclin T1 mRNA levels when cyclin T1 protein expression is up-regulated has also been observed in primary monocytes [24]. The mRNA level of CD11c, a marker for macrophage differentiation that has previously been shown to be induced at the mRNA level[28], increased over 30-fold following the PMA treatment, whereas the mRNA level of CDK9 remained constant (Fig. 1C). Data shown in Figure 1 indicate that the up-regulation of cyclin T1 expression in MM6 cells involves a post-transcriptional mechanism, similar to that observed in primary monocytes. Therefore, MM6 cells appear to be a valid model system with which to investigate the functional significance of cyclin T1 induction during the differentiation of primary monocytes to macrophages.

### Knock-down of cyclin T1 in MM6 cells by a lentiviral shRNA expression vector

To study the functional significance of the induction of cyclin T1 during MM6 differentiation, a siRNA-based strategy was used to knock down cyclin T1 expression. MM6 cells, like many promonocytic cell lines, are refractory to transfection procedures [29] and we therefore used a lentiviral shRNA expression vector. Additionally, the continuous expression of the shRNA from the lentiviral vector in the transduced cells has the advantage of a stable knock-down of cyclin T1 mRNA, while transfected siRNAs typically induce only a transient knock-down [18]. The shRNA expression is driven by the human U6 promoter, a promoter recognized by the RNA polymerase III enzyme [30]. The vector also contains an eGFP expression cassette driven by the human ubiquitin-C promoter. Importantly, the lentiviral vector does not encode any lentiviral gene products. The target sequence for cyclin T1 was selected by a rational design strategy [31]. A control lentiviral vector was constructed in which the shRNA contained a four-nucleotide mismatch against the cyclin T1 mRNA.

**Figure 2:**
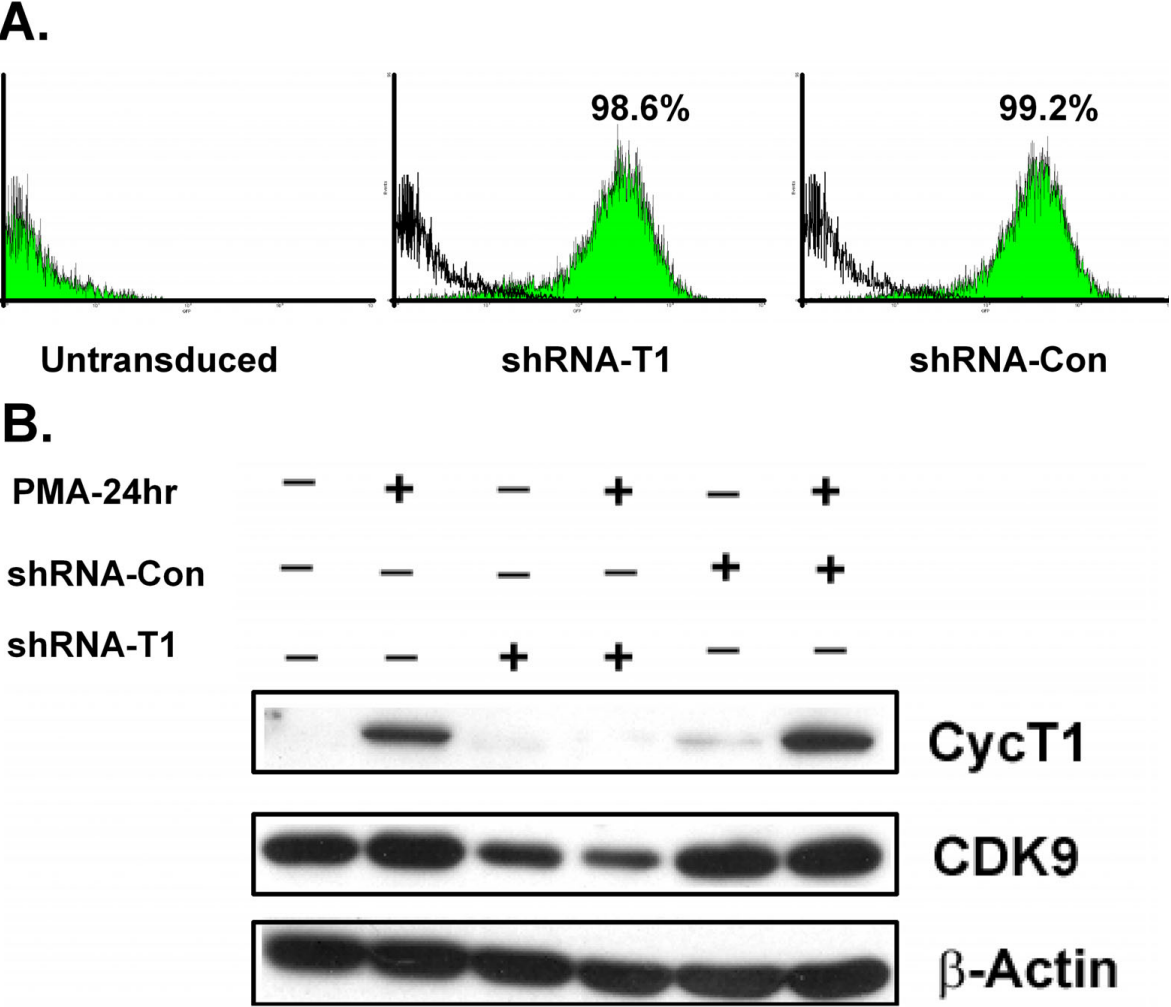
**shRNA against cyclin T1 expressed from a lentiviral vector can efficiently knock down cyclin T1 protein expression**. (A) Untransduced cells (Parental MM6 cells) or MM6 cells infected at a m.o.i. of five with lentiviral vectors expressing a shRNA against cyclin T1(shRNA-T1) or a control shRNA against a mismatch sequence in cyclin T1 (shRNA-Con) were analyzed by flow cytometry at day five post-infection. The lentiviral vectors express an eGFP marker protein. The percentages of the GFP positive cells are indicated. (B) Cell extracts were prepared at day five post-infection from the cultures described in A which were either untreated or treated with PMA for 24 hours. Immunoblots were performed to measure levels of cyclin T1, CDK9 and β-actin proteins.

Using a multiplicity of infection of five, >98% of MM6 cells were transduced five days post-infection with the lentiviral vectors (Fig. 2A). To examine the efficiency of the knock-down, the mRNA and protein levels of cyclin T1 were measured by quantitative RT-PCR and immunoblotting, respectively. The shRNA vector against cyclin T1 reduced cyclin T1 mRNA levels 4-fold relative to parental cells treated with PMA (data not shown). The protein level of cyclin T1 was also significantly knocked down by the cyclin T1 shRNA vector before and after PMA treatment (Fig. 2B). During the course of this study, we observed that CDK9 protein levels were usually reduced when cyclin T1 expression was knocked down by the shRNA vector. For example, the level of CDK9 in the cells infected with shRNA-CycT1 lentivirus was below that of the control cells, both before and after PMA treatment (Fig. 2B). This observation is consistent with previous findings which have indicated that CDK9 protein stability appears to be affected by the expression of cyclin T1 [18].

### Knock-down of cyclin T1 inhibits HIV-1 transactivation by Tat

It is well established that cyclin T1 in the P-TEFb complex is required for Tat-mediated transactivation of HIV-1 LTR-directed gene expression [17]. To test whether the knock-down of cyclin T1 in MM6 cells inhibits the cyclin T1/P-TEFb complex and therefore Tat function *in vivo*, infections were carried out with two HIV-1 luciferase reporter viruses: a virus expressing a wild-type Tat protein and a mutant virus that expresses a non-functional Tat protein. The Tat mutant, Tat-pro18IS has been shown previously to abolish Tat trans-activation [32].

**Figure 3:**
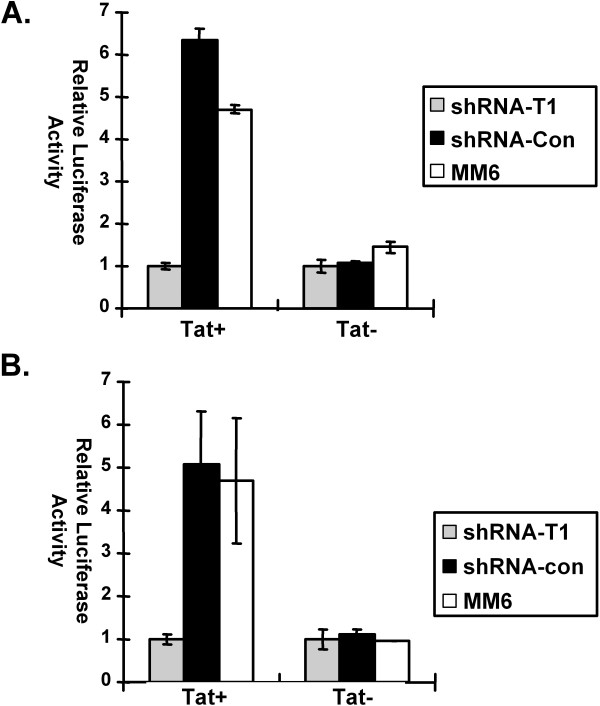
**Knockdown of cyclin T1 inhibits Tat transactivation of HIV-1 proviral expression**. (A) Non-transduced parental MM6 cells (MM6) or pool of MM6 cells expressing a shRNA against cyclin T1 (shRNA-T1) or a control shRNA (shRNA-Con) were infected with either a NL4-3-Luc (Tat+) HIV-1 luciferase reporter virus or a NL4-3-Luc-Tat^- ^(Tat-) virus encoding a mutated Tat protein. Cell lysates were prepared 48 hours post-infection and analyzed for luciferase activity using equal amounts of protein. Luciferase expressions in extracts infected with the shRNA-Cyc T1 lentiviral vector were assigned an arbitrary value of 1.0 unit and other values are shown relative to this. A representative experiment of this experimental design is shown. (B) MM6 cells were infected with either a Tat+ virus or a Tat- virus. After three days, they were either left uninfected or infected with lentivial vectors expressing a shRNA against Cyclin T1 or a control shRNA. Cell extracts were prepared five days post-infection and assayed for luciferase expression. A representative experiment of this experimental design is shown.

Non-transduced MM6 cells or cultures of MM6 cells transduced with shRNA-CycT1 or shRNA-control lentiviruses (five days post-transduction) were infected with either the Tat^+ ^or Tat^- ^reporter virus. For the Tat^- ^virus, luciferase expression was at similar levels in all three infected cultures. However, for the Tat^+ ^virus, luciferase expression was 6-fold lower in cells transduced with shRNA-CycT1 than in non-transduced cells or cells expressing the control shRNA (Fig. 3A). In general, Tat transactivation of the HIV-1 LTR is low in monocytic cell lines relative to Tat transactivations in many other cell lines [24,33].

To exclude the possibility that shRNA against cyclin T1 might affect steps in the virus life cycle prior to transcription of the integrated provirus, MM6 cells were first infected with either the Tat^+ ^or Tat^-^reporter virus. Three days later, the cultures were infected with the lentiviral shRNA vectors. Cell extracts were prepared five days after infection with shRNA vectors and luciferase expression was assayed (Fig. 3B). Again, luciferase expression for the Tat^- ^virus was at similar levels in all three infected cultures. However, for the Tat^+ ^virus, luciferase expression was 5-fold lower in cells infected with shRNA-CycT1 lentiviruses than in non-transduced cells or cells infected with shRNA-control (Fig. 3B). We conclude from these experiments that the shRNA against cyclin T1 is effective in inhibiting cyclin T1 function *in vivo*.

### The knock-down of cyclin T1 in MM6 cells does not affect cell growth

**Figure 4:**
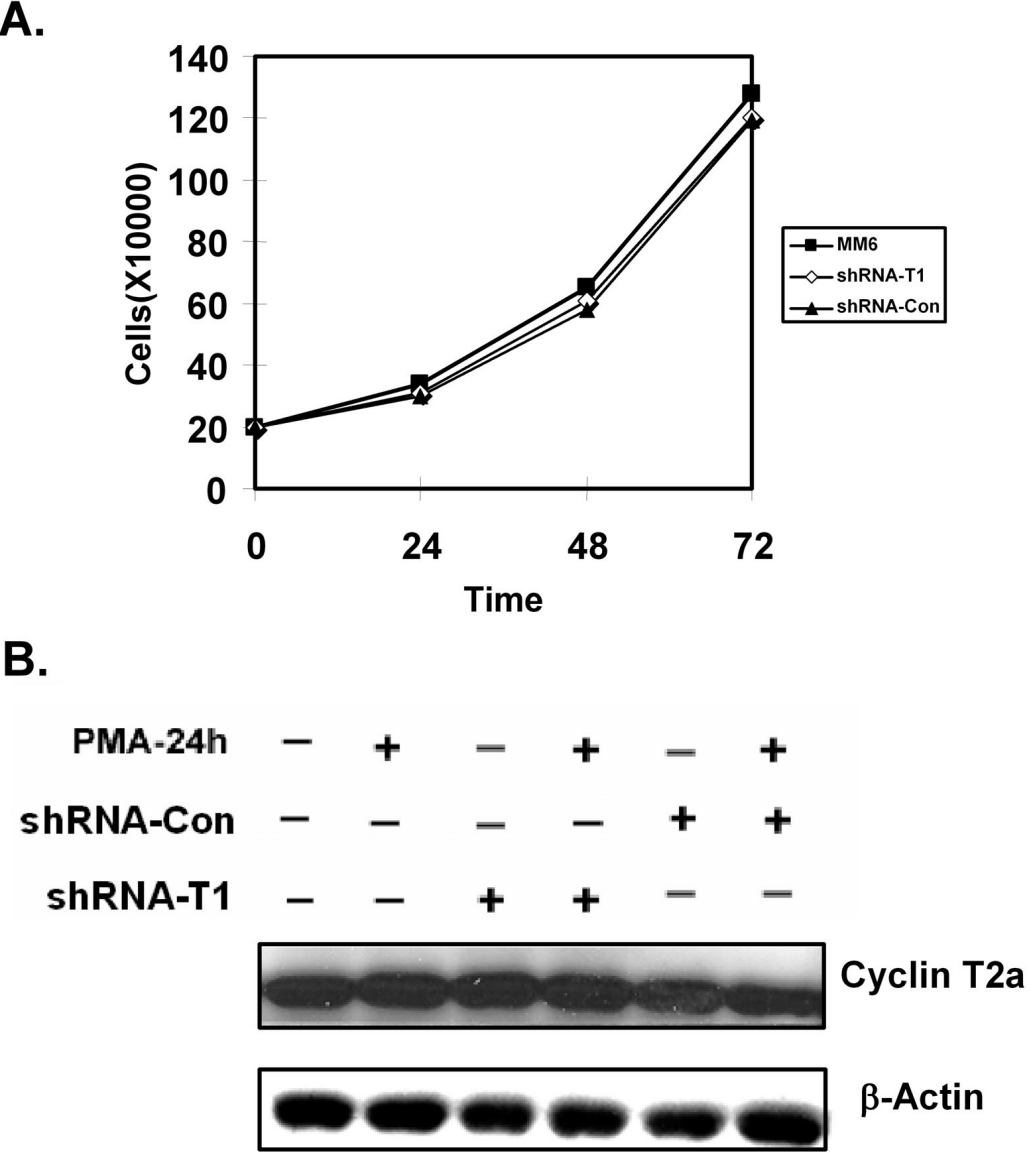
**Cyclin T1 knockdown does not affect cell growth**. (A) Two days after infection with either the shRNA-CycT1 or shRNA-control viruses (T1 and Con), 2 × 10^5 ^cells/ml of the infected or uninfected parental MM6 cell cultures (MM6) were seeded and counted at 24, 48, and 72 hours. (B) Cell lysates were prepared from cells with different treatments (as indicated), five days post-infection. Immunoblots were performed to determine the expression of cyclin T2a and β-actin.

We carried out a growth curve with MM6 cultures two days after infection with the shRNA-CycT1 and shRNA-control lentiviruses. Interestingly, cells expressing the siRNA against cyclin T1 did not exhibit reduced growth, as the culture infected with the shRNA-CycT1 lentivirus grew at a rate equivalent to the culture infected with the shRNA-control virus (Fig. 4A). We observed no increase in spontaneous apoptosis in cells infected with either lentiviral vectors as determined by caspase-3 assays (data not shown). Additionally, no significant difference in the caspase-3 activity was observed in cell extracts prepared from cultures shown in Fig. 4A that were PMA treated (data not shown). The cultures infected with both shRNA-CycT1 and shRNA-control lentiviruses appeared to grow at a slightly reduced rate relative to the parental MM6 cells (Fig. 4A). However, the significance of this small difference is unclear. Additionally, we observed that cells infected with either the shRNA-control or shRNA-CycT1 vector aggregated more than uninfected MM6 cultures prior to PMA treatment, with the shRNA-control vector displaying slightly greater aggregation than the shRNA-CycT1 vector. We did not quantify this phenomenon and its significance remains to be established.

Because the P-TEFb complex includes CDK9 and either cyclin T1, T2a, T2b, or K, it is conceivable that cyclin partners of CDK9 other than cyclin T1 might be sufficient for P-TEFb function in MM6 cells depleted for cyclin T1 expression. We therefore examined cyclin T2a expression in an immunoblot, and a relatively high level of cyclin T2a expression was observed with or without the cyclin T1 knock-down (Fig. 4B). We also observed in immunoblots that cyclin T2b was expressed at low levels in MM6 cells containing the cyclin T1 knock-down (data not shown). Additionally, the expression of cyclin T2a did not change before or after PMA treatment (Fig. 4B). These observations suggest that cyclin T2a and T2b might be responsible for constitutive gene expression in MM6 cells, whereas cyclin T1 might play a more regulatory role in MM6 cells.

### Transcriptional profiling: validation and analysis of microarray data

To identify genes regulated directly or indirectly by cyclin T1 in both PMA-treated and Non-PMA-treated MM6 cells, we performed a transcriptional profile analysis of cultures of MM6 cells infected with the shRNA-CycT1 or shRNA-control lentiviruses, as well as uninfected parental MM6 cells. Cultures were treated with or without PMA and the RNA isolated from these cells were analyzed using Affymetrix human genome U133 Plus 2.0 DNA arrays representing about 18,953 unique (non-redundant) transcripts. Three independent biological replicate experiments were carried out in this analysis. In the first two replicates, all three cultures of cells (parental MM6, shRNA-CycT1, shRNA-control) were treated with or without PMA. In the additional replicate, only cells treated with PMA were analyzed.

**Figure 5:**
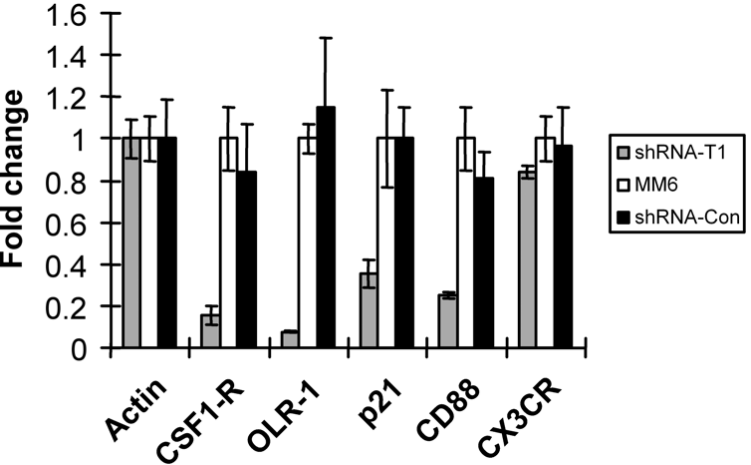
**Validation of the microarray data**. Cell cultures were infected with indicated shRNA lentiviral vectors for five days, treated with PMA for 24 hours, and total RNA was isolated. Quantitative real-time RT-PCR was used to measure the expression level of corresponding mRNA. The fold change represents the change of transcript levels in cells relative to parental MM6 cells after normalization with β-actin levels.

To verify that the microarray data are reliable, several mRNAs whose levels were up-regulated >2-fold by PMA treatment and were also repressed >2-fold by shRNA-CycT1 were selected for further analysis by real-time RT-PCR assays: colony stimulating factor 1 receptor (CSF1-R), oxidised low density lipoprotein (lectin-like) receptor 1 (OLR1), cyclin-dependent kinase inhibitor 1A (p21) and complement component 5 receptor 1 (CD88). Chemokine (C-X3-C motif) receptor 1 (CX3CR1) was selected as a negative control, as its RNA levels was unaffected by the cyclin T1 knock-down in the microarray data. Additionally, RNA levels were normalized to β-actin whose level was unaffected by PMA or knock-down of cyclin T1. The fold-change of transcripts in shRNA-CycT1 cells were compared with the parental MM6 cells (Fig. 5). In excellent agreement with the microarray data, transcripts encoding these genes were also repressed in cells expressing shRNA-CycT1. These data suggest that the microarray data are in general reliable.

Affymetrix microarray data were processed in three steps: 1) normalization and derivation of expression measures; 2) analysis of expression measures with a linear model to identify lists of differentially expressed genes; and 3) content analysis of the gene lists to distill biologically interpretable content. All analyses were conducted in the R open source language for statistical computing using both the Bioconductor suite of R packages and locally developed R code[34].

**Figure 6:**
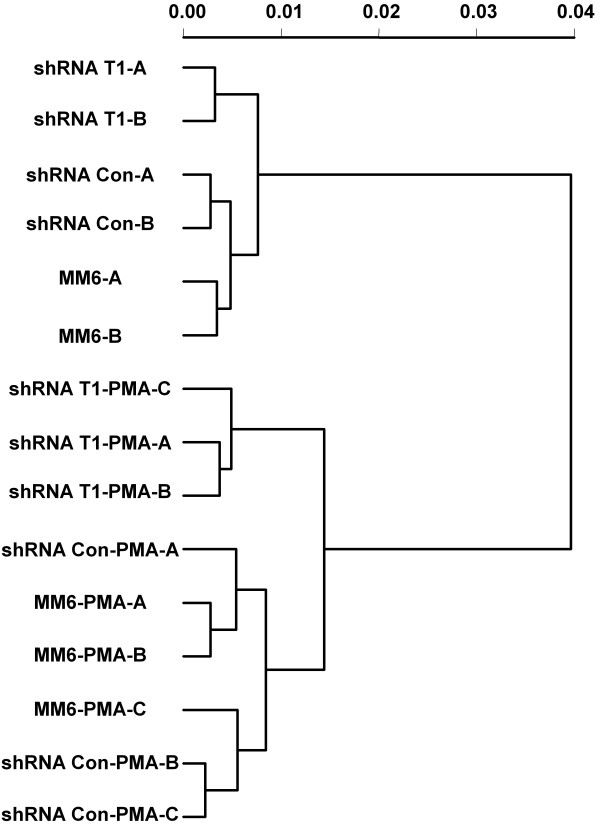
**Cyclin T1 knockdown cells have a distinct gene profile compared to control cells**. A dendrogram was constructed based on the data from all probe sets for all 15 arrays used in the study. The Pearson Correlation distance was calculated to represent the expression differences between the arrays. The leaves of the tree represent each of the 15 arrays used in this study. The branches denote the relative distances between the samples. Branch joins near the leaves of the tree represent high similarity, while deeper joins represent less similarity.

Raw probe level intensity data were reduced to expression measures using the gcrma method [35]. To examine the pattern of differences in RNA populations from cultures subjected to different treatments, a dendrogram was generated based on expression measures from all probe sets on the array (Fig. 6). The 15 RNA samples were clearly partitioned into four groups: 1) shRNA-CycT1 cells without PMA treatment; 2) shRNA-control and parental cells without PMA treatment; 3) shRNA-CycT1 cells with PMA treatment; 4) shRNA-control and parental cells with PMA treatment. This grouping suggests that the knock-down of cyclin T1 has a distinct gene expression profile from that of shRNA-control or parental cells. Additionally, this grouping suggests that the gene expression profiles from the shRNA-control and parental cells are very similar to each other and can be treated as a single control group.

To better understand the genes responsible for the pattern observed in the dendrogram, a two-way ANOVA was fit to each probeset using activation and knockdown state as explanatory variables. A linear contrast analysis was then performed to identify differentially expressed genes (see below). The contrast analysis identified four distinct sets of genes: PMA-induced, PMA-repressed, T1 knock-down-induced-in-PMA-treated-cells, and T1 knock-down-repressed-in-PMA-treated-cells. An empirical Bayes method[36] was used to enhance variance estimation and to improve the T-statistics for individual probe sets. Multiple testing corrections were made using the Linear Step Down method[37]. Lists were formed using the rule that a greater than 2-fold change in expression was estimated between the treatments, and the adjusted false discovery rate (FDR) value for the comparison was less than 0.05.

Finally, the genes identified in the various lists were subjected to gene ontology (GO) content analysis[38]. GO content analysis was performed by tabulating the list against the GO structure. To perform the analysis, we calculated the number of genes in the list annotated at or below each GO node. This number is then compared against the distribution of counts expected for a random list of the same size. Statistical consideration of the counts is based on a sampling without replacement model for counts, treating the entire array as the universe of possible genes from which a random list might be constructed. The results indicated a large and distinctive family of differences between the content of the various lists. The analyzed microarray data can be downloaded from: .

### Cyclin T1 is required for the appropriate expression of a sizable portion of mRNAs regulated by PMA

In our transcriptional profiling data, PMA treatment and cyclin T1 knock-down are two major variables in the RNA samples. The microarray data were therefore analyzed to determine the effects of PMA treatment and knock-down of cyclin T1 on RNA expression in MM6 cells.

We first examined the genes in control cells (no cyclin T1 knock-down) that were either induced or repressed by PMA treatment. These 10 control samples (shRNA-control and parental MM6 cells) were separated into two groups: six PMA-treated samples and four untreated samples. A statistical analysis of these control samples revealed that a set of 1460 genes were upregulated >2-fold by PMA, and 1525 genes were downregulated >2-fold by PMA, with an adjusted FDR value of P < 0.05. Thus, in control cells, 7.7% of genes assayed (1460 of 18,953) were induced >2-fold by PMA, while 8.0% of genes (1525 of 18,953) were repressed >2-fold by PMA (Table 1).

**Table 1:** Number of genes induced or repressed >2-fold by different treatments

	Induced	Repressed
Non-PMA-treated vs PMA-treated (shRNA-con & parental cells)	1460 (7.7%)	1525 (8.0%)
shRNA-T1 vs shRNA con (non-PMA-treated cells)	87 (0.5%)	131 (0.7%)
shRNA-T1 vs shRNA con (PMA-treated cells)	399 (2.1%)	438 (2.3%)

The number of genes that were affected by the depletion of cyclin T1 in cells without PMA treatment was calculated. The two shRNA-CycT1 samples from non-PMA treated cells were compared with two shRNA-control samples. A statistical analysis of these samples revealed that a set of 131 genes were repressed >2-fold in the shRNA-CycT1 samples, and 87 genes were induced >2-fold in the shRNA-CycT1 samples, with an adjusted FDR value of P < 0.05. Thus, in non-PMA treated cells, 0.5% of genes assayed (131 of 18,953) were repressed >2-fold by shRNA-CycT1, while 0.7% of genes (87 of 18,953) were induced >2-fold by shRNA-CycT1 (Table 1).

We next examined the number of genes that were affected by cyclin T1 knock-down in cells treated with PMA. The three PMA-treated shRNA-CycT1 samples were compared with three PMA-treated shRNA-control samples. A statistical analysis revealed that following PMA treatment, a set of 438 genes were repressed >2-fold by the cyclin T1 knock-down, while 399 genes were induced >2-fold by the knock-down (P < 0.05). Thus, in these PMA-treated cells, 2.3% of genes assayed (438 of 18,953) were expressed at lower levels in cyclin T1 knock-down cells, while 2.1% of genes (399 of 18,953) were expressed at higher levels in cyclin T1 knock-down cells (Table 1).

**Figure 7:**
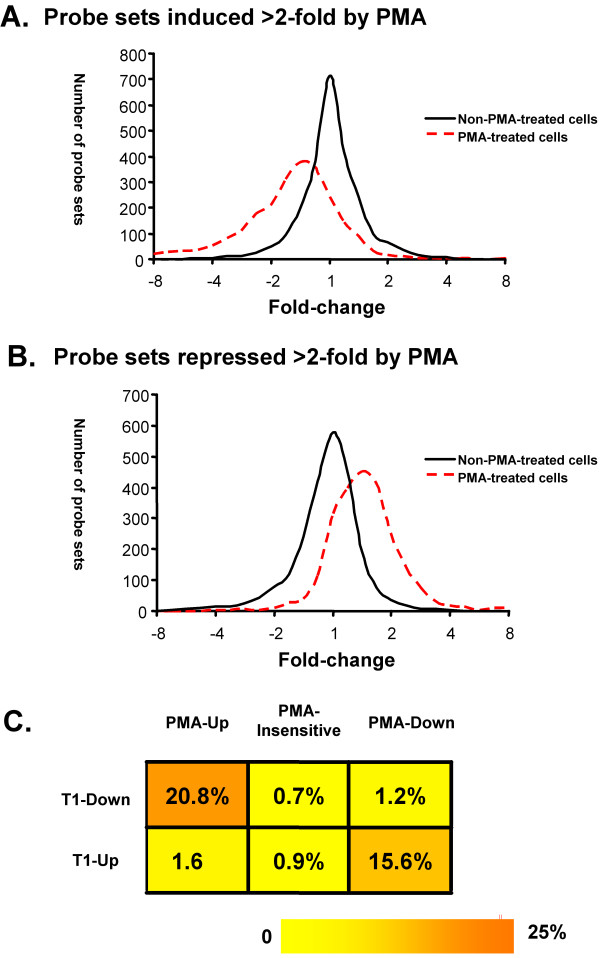
**Cyclin T1 specifically affects genes regulated by PMA**. (A) For probe sets that were induced >2-fold by PMA treatment, fold-change for shRNA-T1 versus shRNA-Con treatment was calculated. Based on magnitude of the fold-change, a total of 26 categories (bins) were created. The first bin included all the probe sets with a fold-change < -8 and the last bin included all the probe sets with a fold-change >8. For bins between -8- and 8- fold (-3 to 3 after log2 transformation), the probe sets were divided into 24 bins with a log2 transformed value of 0.25 as the range for each bin (-3 to -2.75, -2.75 to -2.5, etc.). The number of probe sets in each bin (frequency) was tabulated and is shown on the Y axis. A histogram was generated based on the frequencies in each corresponding bin, representing the distribution of the fold-changes for those probe sets. The black solid line represents the histogram generated from microarray data from non-PMA-treated cells, while the red broken line represents the histogram generated from the microarray data from PMA-treated cells. (B) For the probe sets that were repressed >2-fold by PMA treatment, fold-change for shRNA-T1 versus shRNA-Con treatment in non-PMA-treated or PMA-treated cells was calculated. A histogram was generated as described above based on the distribution of the fold-changes of those probe sets. (C) Genes that were either upregulated or downregulated by cyclin T1 knock-down were divided into three classes: PMA-induced genes (PMA-Up), PMA-insensitive genes, and PMA-repressed genes (PMA-Down). Number of genes in each category was calculated from the microarray data. Those numbers were then divided by the total numbers for the PMA-induced genes, PMA-repressed genes, or PMA-insensitive genes to obtain the corresponding percentage.

To examine globally how the set of PMA-regulated genes in MM6 cells are affected by the knock-down of cyclin T1, we examined the effect of the knock-down on probe sets that were either induced or repressed >2-fold by PMA treatment in parental and shRNA-control cells. For every probe set, its fold-change in shRNA-CycT1 versus shRNA-control was calculated, with a negative score representing downregulation by the knock-down and a positive score representing upregulation by the knock-down, A histogram was then generated based on the distribution of the scores of all the probe sets that were either upregulated or downregulated by PMA (Fig. 7). We examined the effect of cyclin T1 on gene expression in untreated cell and PMA-treated cells separately. In untreated cells, most probe sets had scores between -2 and 2, suggesting that cyclin T1 has little effect (<2-fold) on the set of PMA-regulatable genes (Fig. 7). We do note, however, that in non-PMA-treated cells the cyclin T1 knock-down induced a small number of PMA-upregulated genes >2-fold, suggesting a very low level of activation occurred following the shRNA-CycT1 lentivirus infection (Fig. 7A). In PMA-treated cells, an obvious shift was observed in the distribution of the fold-changes caused by the cyclin T1 knock-down in those PMA-regulatable genes. For genes that are PMA-inducible, a leftward shift was observed and a sizeable number of genes were downregulated >2-fold by knock-down of cyclin T1 (Fig 7A). For genes that are PMA-repressed, a rightward shift was observed and a sizeable number of genes were upregulated more than >2-fold by knock-down of cyclin T1 (Fig. 7B). Overall, these data indicate that the level of induction of a significant fraction of PMA-inducible genes is repressed by cyclin T1 depletion, and likewise, the level of repression of a significant fraction of PMA-repressed genes is induced by cyclin T1 depletion.

To quantify the minimum number of PMA-regulated genes affected by the knock-down of cyclin T1, the list of genes affected by cyclin T1 knock-down were compared to the list of genes affected by PMA treatment in the control group (Fig. 7C). We found that 303 of 1460 (20.8%) PMA-inducible genes were repressed by cyclin T1 knock-down. In contrast, <1% of the PMA-insensitive genes and 1.2% of the PMA-repressed genes were repressed by cyclin T1 knock-down. Similarly, 238 of 1525 (15.6%) PMA-repressed genes were expressed at higher levels in cyclin T1 knock-down cells. In contrast, <1% of the PMA-insensitive genes and 1.6% of PMA-inducible genes, were induced by cyclin T1 knock-down. This observation strongly suggests that cyclin T1 specifically modulates expression of a substantial fraction of genes that are regulated by PMA. Our data suggests that the induction of cyclin T1 in PMA-treated cells contributes to the induction of a minimum of 21% of PMA-inducible genes and a minimum of 16% of PMA-repressed genes.

### Genes involved in immune response are over-represented in the set of genes affected knock-down of cyclin T1

A Gene Ontology analysis was performed to identify the biological processes mediated by genes induced in PMA-treated MM6 cells. GO provides an organized vocabulary of terms that can be used to describe a gene product's attributes [39]. For the group of genes included in each GO term, a significance value is computed from the microarray data. This value (P value) is used to identify biological processes that are either over-represented or under-represented in those RNAs whose expression levels are altered by different conditions.

**Figure 8:**
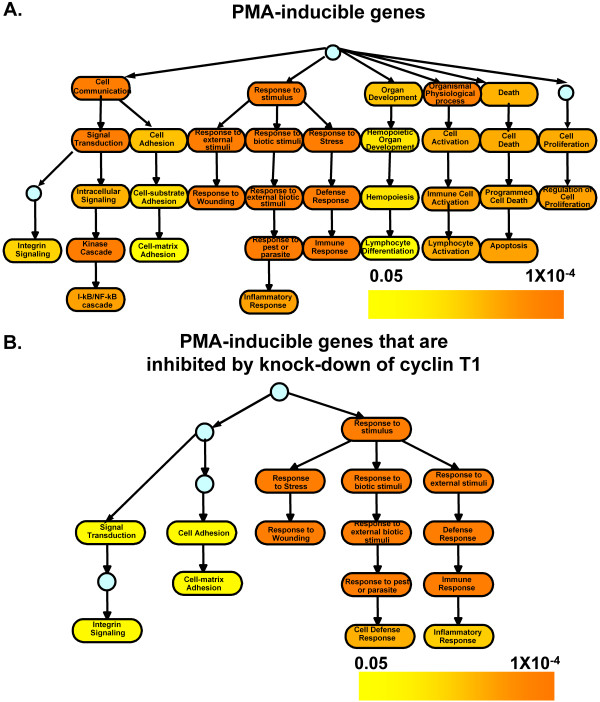
**Gene Ontology analysis**. GO content analysis was performed by tabulating the various gene lists against the GO structure. (A) Biological processes that were over-represented in the PMA-inducible genes compared to a random list of the same size. The color of each GO term was assigned corresponding to its adjusted P-value, while color intensity representing the adjusted P-value is indicated by color scaling. (B) Similar analysis was performed on genes induced by PMA and repressed by shRNA-T1. The color of each GO term was assigned corresponding to its adjusted P-value, while color intensity representing the adjusted P-value is indicated by color scaling.

For genes induced by PMA in control samples, the over-represented biological processes were largely related to immune responses, signal transduction, cell proliferation and apoptosis (Fig. 8A). This pattern was expected, as PMA induces a program of macrophage differentiation in MM6 cells, and these biological processes are known to affect macrophage function and the differentiation program.

We next performed a GO analysis for the PMA-inducible genes that were inhibited by the knock-down of cyclin T1 (Fig. 8B). A GO profile was obtained that was related but nonetheless distinct from that seen in PMA-induced genes. The GO terms that are related to cell proliferation, cell cycle and apoptosis seen in the PMA-inducible genes were not over-represented in the genes that were affected by cyclin T1 knock-down. A comparison between those two GO analyses (data not shown) revealed that the GO terms related to immune response were more significantly over-represented in the genes inhibited by knock-down of cyclin T1 than in control PMA-treated cells. This suggests that the knock-down of cyclin T1 may specifically affect genes related to the immune response.

## Discussion

Although the induction of cyclin T1 is observed during early macrophage differentiation, its functional significance was unknown prior to this study, due to the difficulty in biochemical and genetic manipulation of primary monocytes. In this study, we used MM6 cells as a model system to study the regulation and function of cyclin T1 during the monocyte differentiation process. Cyclin T1 was induced in MM6 cells upon PMA treatment by a post-transcriptional mechanism, similar to its induction in primary monocytes and macrophages. Although the knock-down of cyclin T1 in MM6 cells by shRNA inhibited HIV-1 Tat transactivation, MM6 cell growth was not affected by the depletion of cyclin T1. Using DNA microarray technology, we found that more than 20% of genes induced by PMA require cyclin T1 for their normal level of induction, and more than 15% of genes repressed by PMA require cyclin T1 for their normal level of repression. Gene ontology analysis indicates that a high portion of these cyclin T1-dependent genes are related to the immune response and signal transduction. These results suggest that cyclin T1 serves a critical role in the program of macrophage differentiation.

We found that cyclin T1 protein expression is induced by a post-transcriptional mechanism after the treatment of MM6 cells by several different differentiation inducers and activators (Fig. 1). These observations in MM6 cells are similar to our previous findings in primary human monocytes, where the induction of cyclin T1 early during differentiation to macrophages also occurs by a post-transcriptional mechanism. Furthermore, a gene ontology analysis of mRNAs induced in MM6 cells by PMA demonstrated an over-representation of genes that are related to the immune response, signal transduction, cell growth and apoptosis – biological processes known to be involved in monocyte and macrophage differentiation or function (Fig. 8). Our data indicate that MM6 cells are a valid model system with which to study the functional role of cyclin T1 during macrophage differentiation.

To our surprise, the stable knock-down of cyclin T1 in MM6 cells did not measurably affect cell growth (Fig. 4), although it did result in an inhibition of Tat-dependent HIV-1 gene expression (Fig. 3), indicating that cyclin T1/P-TEFb function was reduced. In non-PMA-treated MM6 cells, the great majority of cellular genes were not affected by the depletion of cyclin T1, as only 131 genes (0.7% of the genes analyzed) were repressed >2-fold and 87 genes (0.5%) were induced >2-fold. The high level of cyclin T2a, an alternative cyclin partner of CDK9 in MM6 cells, might be sufficient to sustain adequate P-TEFb levels and therefore general gene transcription elongation following the cyclin T1 knock-down. In MM6 cells, it is possible that cyclin T1 and T2 have largely redundant functions and the knock-down of cyclin T1 may be almost fully compensated by cyclin T2a (or T2b). Alternatively, because cyclin T1 is expressed at a low level in the non-activated MM6 cells, it may play a more regulatory role, while cyclin T2 may be responsible for expression of the set of constitutively expressed genes in monocytic cells. In agreement with this, we have recently observed that cyclin T2a is also expressed constitutively in primary human monocytes and is not induced by differentiation or macrophage activation [26].

Although non-PMA-treated MM6 cells with the knockdown of cyclin T1 grow well, cyclinT1 expression appears to be required for the regulated expression of a sizeable portion of PMA-regulated genes, both those inducible and repressed by PMA treatment (Fig. 7). Greater than 20% of genes induced by PMA require cyclin T1 for their normal level of induction, while greater than 15% of genes repressed by PMA require cyclin T1 for their normal level of repression. Therefore, cyclin T1 is likely to play an important role in MM6 differentiation. It is important to note that our data does not address whether the genes affected by the depletion of cyclin T1 are direct targets of cyclin T1/P-TEFb, rather than the results of indirect effects that arise from a cascade of gene expression. Nonetheless, it is clear that the knock-down of cyclin T1 preferentially affects the expression of PMA-regulated genes rather than a representative subset of all genes expressed in non-stimulated MM6 cells.

The cyclin T1 knock-down specifically affects the mRNA levels of many genes related to the immune response and signal transduction in MM6 cells. In late-differentiated primary macrophages, cyclin T1 expression is shut off by proteasome-mediated proteolysis and can be reinduced with activation by LPS or other PAMPs. The re-induction of cyclin T1 in macrophage by PAMP activation suggests that cyclin T1 may have an important role in the innate immune response. It seems likely that there is considerable overlap between genes in MM6 cells that are affected by the knock-down of cyclin T1 and the genes affected by the re-induction of cyclin T1 in late-differentiated macrophages. It is notable that HIV-1 can induce the expression of cyclin T1 protein in primary macrophages, and therefore hijack this component of an innate immune response to enhance viral replication [25].

Tat transactivation is highly dependent on cyclin T1/P-TEFb function, and inhibitors of this cellular function, such as small molecules, a dominant CDK9 protein, anti-hCycT1 intrabodies, and siRNA against P-TEFb, are effective in reducing HIV-1 replication *in vitro *[18-23]. We have shown that the knockdown of cyclin T1 in MM6 cells has a pronounced effect on Tat transactivation during HIV-1 infection. The cyclin T1 depletion described here, similar to previous studies in HeLa or 293T cells, did not measurably affect the growth of MM6 cells. However, the cyclin T1 knockdown preferentially affected PMA-regulated genes and therefore it is likely that the depletion of cyclin T1 in primary macrophages will affect the innate immunity and macrophage activation. Therefore, the use of shRNA or siRNA to deplete cyclin T1 and reduce Tat function may not be feasible as an antiviral therapeutic strategy.

Finally, identification of genes regulated by cyclin T1 may eventually help identify novel cellular factors important for HIV-1 replication. Cyclin T1 in the P-TEFb complex is required for Tat-mediated transactivation of HIV-1 LTR-directed gene expression. It is possible that the induction of cyclin T1 and the consequent upregulation of cyclin T1-dependent genes may provide a permissive environment of HIV-1 replication. Therefore, by using the cyclin T1/P-TEFb complex as a cofactor, the virus may assure an optimal cellular environment for maximal virus production. Further analysis of the genes regulated by cyclin T1 may lead to the identification of additional cellular factors important for HIV-1 replication.

## Conclusion

HIV targets the cyclin T1/P-TEFb complex to mediate Tat transactivation. Cyclin T1 is also induced during monocyte differentiation. The functional significance of cyclin T1 was studied in MM6 cell as a model system for monocyte differentiation. Although cell growth was not affected by depletion of cyclin T1, cyclin T1 seems to play an important role in regulating a large portion of mRNA related to the differentiation program. This raises questions about using cyclin T1 as a therapeutic target for HIV-1 infection.

## Methods

### Cell culture and reagents

Mono-Mac-6 cells were a gift from Dr. Jorge Benach (State University of New York at Stony Brook). Cells were maintained in RPMI 1640 medium (Invitrogen) supplemented with 10% heat-inactivated fetal bovine serum (FBS, Hyclone), non-essential amino acids (Invitrogen), l-glutamine (Invitrogen), and OPI media supplement (Sigma) containing 0.15 mg of oxalacetate, 0.5 g of pyruvate and 8.2 mg of bovine insulin. For activation experiments, MM6 cells were treated at a final concentration of 10 ng/ml phorbol 12-myristate 13-acetate (PMA, Sigma), 50 nM vitamin D3 (Sigma), 25 uM retinoic acid (Sigma), 1 ng/ml lipopolysacchride (LPS, Sigma) and 500 U/ml interferon gamma (R&D systems).

### Cell extracts and immunoblotting

Cell extracts were prepared by incubating cells in lysis buffer (50 mM Tris, 120 mM NaCl, 0.5% NP-40) containing protease inhibitors (2 μg/ml aprotinin, 1 μg/ml leupeptin, 2.5 mM phenylmethylsulfonyl fluoride) as described previously [33]. Protein concentrations were determined by a Bio-Rad protein assay, and 20 μg of total protein was loaded onto sodium dodecyl sulfate-9% polyacrylamide gels. The procedure for immunoblots using enhanced chemiluminescence for detection has been described previously[40]. Antibody to β-actin was purchased from Sigma, and other antibodies were purchased from Santa Cruz Biotechnology.

### shRNA design, Lentivial production and flow cytometry

The target sequences used for this study were: shRNA-CycT1: GCAGCGTCTTAACGTCTCA; shRNA-Control: GCTATAGCTGTTCTAGTTC. Oligo-nucleotides containing the target sequences with overhangs compatible with restriction enzyme sites were purchased from Invitrogen. The annealed oligonucleotides were inserted into a hU6-1 plasmid vector immediately after the human U6 promoter. The U6 promoter driven-shRNA expressing cassettes were then subcloned into the FG12 lentiviral vector. The FG12 vector is a self-inactivated lentiviral vector carrying an eGFP expression-cassette; the vector does not encode any viral gene products [41].

Stocks of the FG12 lentiviral vectors pseudotyped with vesicular stomatitis virus (VSV)-G were produced by calcium phosphate-mediated transient transfection of HEK-293T cells. Briefly, HEK-293T cells were cultured in DMEM (GIBCO Invitrogen) containing 10% FBS (HyClone), 100 units of penicillin, and 100 μg/ml streptomycin. The cells were cotransfected with 5 ug of each plasmid: vector plasmid, the HIV-1 lentiviral packaging constructs pRSV/REV and pMDLg/pRRE, and the VSV-G expression plasmid pHCMV-G. Virus stocks were collected from the culture supernatants on days two post-transfection and were titered on HEK-293T cells based on GFP expression. MM6 cells (2 × 10^5^/ml) were transduced at a multiplicity of infection (m.o.i.) of five in the presence of 5 ng/ml polybrene (Sigma).

To determine transduction efficiencies, five days after lentiviral infection cells were suspended at 1 × 10^6 ^cells/ml in phosphate-buffered saline (PBS) with 2% FBS and the percentage of GFP positive cells were determined by flow cytometry using a Beckman-Coulter XL-MCL cytometer.

### HIV-1 luciferase virus production and luciferase assay

The HIV-1 reporter virus NL4-3-Luc (Tat^+^) was pseudotyped with VSV-G envelope protein and contains the firefly luciferase gene in place of Nef. A Tat^- ^NL4-3-Luc reporter virus (Tat^-^) was generated by introducing an EcoR I restriction enzyme site after proline 18 in the Tat coding sequence, which abolishes Tat transactivation function[32]. Stocks of Tat^+ ^and Tat^- ^NL4-3-Luc viruses were produced by calcium phosphate-mediated transient transfection of HEK-293 T cells, with cotransfection of the VSV-G expression plasmid pHCMV-G and the Tat expression plasmid pCMV-Tat. To assay for luciferase production, cells lysates were prepared with Cell Culture Lysis Buffer (Promega). The luciferase assay was performed according to the manufacturer's protocol (Promega), and the products were measured by a luminometer (Turner).

### Microarray analysis

Microarray analysis was performed by the Baylor Microarray Core Facility (Baylor College of Medicine, Houston, TX 77030, USA). Detailed protocols can be found at the website: . Briefly, RNA was isolated using the Qiagen RNeasy kit according to the manufacturer's protocol and RNA quality was determined using an Agilent 2100 Bioanalyzer. RNA was reverse transcribed to generate cDNA and transcribed using T7 RNA polymerase and biotinylated ribonucleotides to generate labeled cRNA. Fragmented cRNA was hybridized to U133 plus 2.0 human gene chips (Affymetrix) containing nearly 55,000 probe sets representing over 18,953 transcripts. Following washing and staining, the arrays were scanned using an Affymetrix Gene Chip Scanner 3000. For all experiments, the 5'/3' ratios of GAPDH ranged between 0.85 and 0.91. Comparisons of matched control and were performed for each of three independent experiments.

### Realtime PCR analysis

Quantitative real-time RT-PCR was performed using the Bio-Rad MyIQ single color detection system. Cellular RNA was used to perform cDNA synthesis using the iScript cDNA synthesis kit (Bio-Rad). Briefly, 1 μg of RNA was reverse transcribed in a 20-μl reaction volume using the manufacturer's protocol. PCR reactions were performed using 3 μl of cDNA in a 50 μl reaction containing 25 μl of 2X iQ SYBR Green Supermix (Bio-Rad) and 200 nM final concentration of each primer. PCR reactions were carried out in 96-well format using a Bio-Rad iCycler with a 3 min hot start followed by 40 cycles of 15 s at 95°C, 1 min annealing and amplification at 55°C. Analysis was performed using the MyIQ software program (Bio-Rad). The threshold crossing (Ct) value for each reaction was determined and the fold-change (ΔΔCt value) was calculated with the following formulas using GAPDH as a reference control:

Primers for quantitative PCR were designed using Beacon Designer 2.0 (Premier Biosoft). All primer pairs produced single amplification products as determined by gel electrophoresis as well as melt-curve analysis using the MyIQ system. Primers used were (5' to 3'): β-actin (forward): AGCAAGCAGGAGTATGACGAGTC, β-actin: AGAAAGGGTGTAACGCAACTAAGTC (reverse), CSF1R(forward): TTCTGCTGCTCCTGCTGGTG, CSF1R(reverse): ACCGTTGCTCCTGGCTTCAC, LOX1(forward): ACTGTGAAGGACCAGCCTGATG, LOX1(reverse): CCTAGAGTCGCAGCAGCCAG, CD88(forward): TCAAGGTGGTGGTGGCAGTG, CD88(reverse): GTGACGATGGCTCCAGGAAGG, P21(forward): AGCAGCGGAACAAGGAGTCAG, P21(reverse): GCCCTGTCCATAGCCTCTACTG, cyclin T1 (forward) AACCTTCGCCGCTGCCTTC, cyclin T1 (reverse) ACCGTTTGTTGTTGTTCTTCCTCTC, Cdk9 (forward) AGCACCAACTCGCCCTCATC, Cdk9 (reverse) TTCAGCCTGTCCTTCACCTTCC.

## Competing interests

The author(s) declare that they have no competing interests.

## Authors' contributions

WY performed the experiments in MM6 cells, performed the analysis of microarray data, and wrote the manuscript. YW and XQ constructed and characterized the HIV-1 viruses and lentiviral vectors used in the study. CS performed the analysis of DNA microarray data and performed the statistical analysis. AR conceived of the study, participated in its design, and wrote the manuscript. All authors read and approved the final manuscript.
